# Adverse Reactions to Taxanes in Premedicated Cancer Patients: A Prospective Observational Study

**DOI:** 10.7759/cureus.89590

**Published:** 2025-08-07

**Authors:** M. B Febina, Princy L Palatty, Keechilat Pavithran, Mahesh K D.

**Affiliations:** 1 Department of Pharmacology, Amrita Institute of Medical Sciences and Research Center, Amrita Vishwa Vidhyapeetham, Kochi, IND; 2 Department of Pharmacology, Amrita Institute of Medical Sciences and Research Center, Amrita Vishwa Vidhyapeetham, Ernakulam, IND; 3 Department of Medical Oncology, Amrita Institute of Medical Sciences and Research Center, Amrita Vishwa Vidhyapeetham, Kochi, IND

**Keywords:** adverse reactions, cancer, infusion reaction, pre medication, taxane

## Abstract

Introduction: Taxanes, including paclitaxel, docetaxel, and cabazitaxel, are widely used anticancer agents that disrupt cell division by binding to microtubules, but are associated with significant adverse reactions, particularly infusion-related reactions (IRRs), such as flushing, urticaria, and respiratory symptoms. Despite premedication with steroids, antihistamines, and antiemetics per guidelines, taxane-induced side effects remain prevalent and can result in treatment delays or discontinuation, impacting patient outcomes. This study aimed to observe and document the incidence and spectrum of adverse reactions to taxanes among premedicated cancer patients to improve management and overall chemotherapy success.

Objectives: To estimate the frequency of adverse reactions to taxanes (paclitaxel, docetaxel, and cabazitaxel) in premedicated cancer patients and assess the causality, severity, and nature of these observed events.

Methodology: Our study was a prospective observational study conducted over six months (July 2023-December 2023). Patients receiving docetaxel, paclitaxel, or cabazitaxel and visiting the Medical Oncology Department of the Amrita Institute of Medical Sciences and Research Center, Kochi, were selected for the study. Patients were monitored for IRRs and other adverse effects during and after the infusion of these drugs. Premedication was administered to all patients before the initiation of chemotherapy. Adverse reactions were recorded and graded according to CTCAE version 5.0 (Common Terminology Criteria for Adverse Events). Causality was assessed by the World Health Organization-Uppsala Monitoring Center (WHO-UMC) system.

Results: A total of 206 cycles were observed in 56 patients. A total of 236 adverse reactions were recorded, out of which nine were IRRs. The most common non-infusion-related adverse reactions were alopecia, numbness, myalgia, and mucositis. The most commonly observed IRRs were flushing, chest tightness, and dyspnea. Both types of adverse reactions showed a severity of less than or equal to grade 2 in the majority of subjects. These adverse effects, which commonly occur during the initial couple of chemotherapy cycles, were mostly deemed to be probable/possible reactions, as per the WHO/UMC causality assessment scale.

Conclusions: IRRs accounted for only a small number of adverse events (3.8%) due to taxanes in premedicated cancer patients, and neither these reactions nor non-IRRs led to the discontinuation of therapy. Integrating desensitization protocols and the Paclitaxel-Hypersensitivity Reaction (Pac-HSR) scoring system into standard practice could further improve the management of IRRs/HSRs in patients on taxane therapy, ensuring better treatment continuity while maintaining high safety standards.

## Introduction

Taxanes are a class of anticancer drugs that act by binding to tubulins/microtubules and play a key role in cell division. Taxanes were discovered by Dr. Monroe Eliot Wall and Dr. Mansukh C. Wani in the 1960s [1]. For many years, taxanes have remained the first-line regimen for the treatment of various cancers such as breast, prostate, stomach, ovary, and lung cancers [2]. The most commonly prescribed taxanes are paclitaxel and docetaxel. Increased use of these drugs can lead to a high incidence of adverse reactions.

Paclitaxel (Taxol) is a natural compound that was originally isolated from the bark of the Pacific yew tree (Taxus brevifolia). Solubilizer and emulsifying agent used for paclitaxel intravenous injection was Cremophor EL (polyoxyethylated castor oil), which was a suspected cause of hypersensitivity reactions [3]. Nanoparticle albumin-bound paclitaxel (nab-paclitaxel) is an innovative formulation that eliminates the need for Cremophor EL. Instead, it employs human serum albumin to encapsulate the hydrophobic paclitaxel molecules into particles approximately 130 nm in size [3].

Docetaxel was obtained from a semisynthetic process using the paclitaxel precursor 10-deacetylbaccatin III extracted from the needles of Taxus baccata. The structure of docetaxel is similar to paclitaxel, but it is more soluble. Cabazitaxel is a semisynthetic taxane derived from the precursor 10-deacetylbaccatin III, which was developed to overcome tumour drug resistance to paclitaxel and docetaxel. Adverse reactions to taxanes include both infusion-related reactions (IRR) and non-IRRs. IRR is an adverse effect that occurs during or within 24 h of infusion. It is non-dose related, unpredictable, generally unrelated to the pharmacological activity of the drug, and they usually resolve when treatment is terminated [4]. These reactions can be true allergic responses (immune-mediated reactions such as anaphylactic reactions) and non-allergic reactions. IRRs related to taxanes most commonly include flushing, urticaria, chest tightness, dyspnea, and abdominal symptoms [5]. Other adverse reactions related to taxanes are myalgia, neuropathy, mucositis, alopecia, nail disorders, neutropenia, and thrombocytopenia [6]. According to National Comprehensive Cancer Network (NCCN) and American Society of Clinical Oncology (ASCO) guidelines, premedication before chemotherapy can reduce the incidence and severity of certain infusion-related adverse reactions [6]. Premedications are prescribed to the patients 30 minutes to 1 hour before the administration of taxanes [6]. The most commonly used premedications are steroids, antihistamines, antiemetics, and antacids. Premedications include intravenous or oral dexamethasone, Diphenhydramine, ondansetron, and ranitidine [7].

In previous studies, paclitaxel and docetaxel have been shown to produce adverse reactions in premedicated patients [5]. These adverse effects were observed both during and after the infusion. Preventing adverse events plays an important role in patient treatment because the serious consequences of these adverse events would affect the quality of life of patients and lead to treatment delay and discontinuation, thus preventing the success of cancer chemotherapy. Thus, our study aimed to observe and document the adverse reactions to taxanes in premedicated cancer patients.

## Materials and methods

Our study was a prospective observational study conducted over a period of six months (July 2023-December 2023). Patients receiving docetaxel, paclitaxel, or cabazitaxel and visiting the Medical Oncology Department of the Amrita Institute of Medical Sciences and Research Center, Kochi, were selected for the study. After obtaining institutional ethics committee approval and written informed consent, patients were monitored for adverse reactions during and after drug infusion. Inclusion criteria for the study were (1) adult cancer patients diagnosed with solid tumors or hematologic malignancies; (2) patients scheduled to receive, or currently receiving, taxane-based chemotherapy with standard premedication regimens as per institutional protocol; and (3) patients able and willing to provide informed consent. Exclusion criteria for the study were (1) patients with prior severe hypersensitivity reactions or anaphylaxis to taxanes requiring permanent drug discontinuation and (2) the presence of serious, uncontrolled comorbidities. All relevant data related to adverse drug events, including patient demographics, concomitant medications, and comorbidities, were noted from the case sheets and the patients vis-à-vis direct conversation. Patients were followed up for at least three cycles, and the documented adverse drug reactions were graded according to CTCAE version 5.0 (Common Terminology Criteria for Adverse Events), and the causality of the adverse events was assessed using the World Health Organization-Uppsala Monitoring Center (WHO-UMC) system [8].

Sample size was calculated based on the proportion of infusion-related reactions reported in a previous study by Tangsaghasaksri and Jainan, where 4.24% (48 out of 1,132) patients experienced infusion reactions to Taxanes. Based on this proportion, with 20% relative precision and 95% confidence, the minimum sample size was estimated to be 40 [9].

## Results

Fifty-six patients, with a mean age of 56 ± 10 years, who were prescribed paclitaxel, docetaxel, or cabazitaxel, were monitored over a total of 206 cycles, with each patient observed for a minimum of three cycles.

In our study, the most commonly prescribed taxane was paclitaxel, administered to 35 patients (62.5%), followed by docetaxel in 20 patients (35.7%) and cabazitaxel in 1 patient (1.8%). Of these, paclitaxel was administered alone in 23 (41.7%) patients, docetaxel in 12 (21.4%), and cabazitaxel in 1 (1.8%). Additionally, paclitaxel combined with carboplatin was administered to 13 (23.2%) patients, docetaxel combined with carboplatin to 5 (8.9%) patients, and docetaxel combined with cyclophosphamide to 2 (3.6%) patients (Table 1). The most commonly treated cancers were breast (29, 51.8%), ovarian (10, 17.9%), and endometrial (5, 8.9%). Other cancers included esophageal (2, 3.6%), lung (2, 3.6%), gastric (2, 3.6%), and prostate (2, 3.6%).

**Table 1:** Number of patients receiving chemotherapy regimens, including single-agent taxane treatments and combination therapies.

Medication	No. of patients (%)
Only paclitaxel	23 (41.7%)
Only docetaxel	12 (21.4%)
Only cabazitaxel	1 (1.8%)
Paclitaxel + carboplatin	13 (23.2%)
Docetaxel + carboplatin	5 (8.9%)
Docetaxel + cyclophosphamide	2 (3.6%)

**Figure 1:**
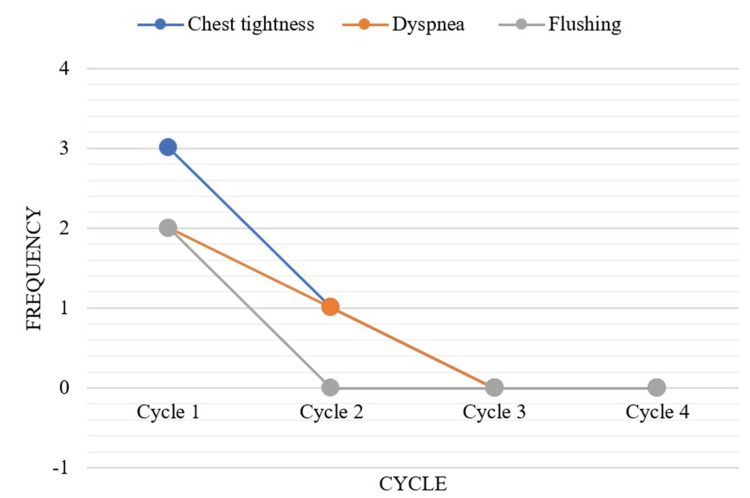
The graph illustrates the incidence of common infusion-related reactions, including chest tightness, dyspnea, and flushing, associated with taxane administration over four cycles, with a higher frequency observed during the initial cycles.

A total of 236 adverse drug reactions were reported across different cycles, and IRRs accounted for only 3.8% (nine patients) of these events. The observed IRRs were chest tightness (4/9, 44.4%), dyspnea (2/9, 22.2%), and flushing (3/9, 33.4%), and these reactions were observed more frequently during the initial couple of chemotherapy cycles (Figure 1). As these reactions were of grade 2 severity, none required permanent discontinuation of the taxane therapy. 

Non-IRR adverse events were classified as dermatologic, gastrointestinal, hematologic, or miscellaneous.

**Figure 2:**
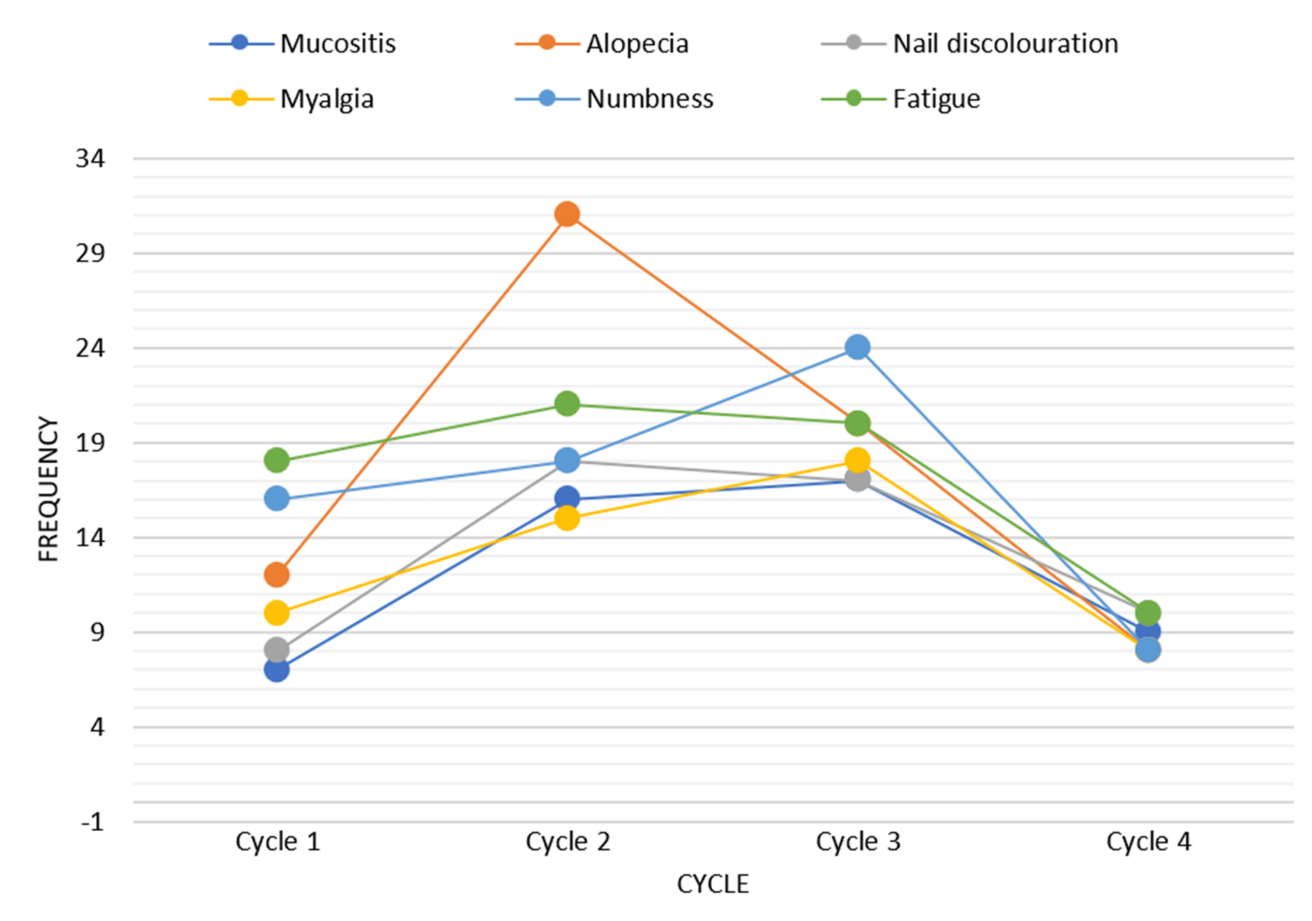
The graph depicts the incidence of common non-infusion-related reactions associated with taxane administration over four cycles, with a higher number of adverse reactions being observed during the second and third cycles.

While the dermatological adverse events observed were alopecia (40, 71.4%), nail discoloration (16, 28.5%), mucositis (14, 25%), and palmar plantar pigmentation (11, 19.6%), the gastrointestinal adverse events included nausea and vomiting (12, 21.4%), diarrhea (5, 8.9%), and constipation (15, 26.7%). Hematological adverse events, such as anemia, were reported in 9 (19.4%) patients, and thrombocytopenia in 1 (2%) patient receiving taxanes (Table 2). Although grade 2 neutropenia occurred in 20 (35.7%) patients, all were successfully managed with filgrastim (granulocyte-macrophage colony-stimulating factor (GM-CSF)) administered every third day during the first two treatment cycles, allowing all patients to complete the full course of therapy. Only one patient experienced grade 3 neutropenia, for which hospital admission was mandatory. The miscellaneous adverse events were neuropathy (31, 58.7%), fatigue (30, 49%), and myalgia (25, 45.1%).Despite being statistically insignificant, peripheral neuropathy was observed more frequently in patients with diabetes mellitus(*P *= 0.5), and tiredness was expressed more frequently in patients receiving concomitant chemotherapeutic agents (*P *= 0.8). For determining the association, the chi-square test was used. Most of the non-IRRs occurred during cycles 2 and 3 (Figure 2), with a severity grading of 2 in approximately 60% of the cases, and a causality assessment was probable for 78% of taxane-induced non-IRRs.

**Table 2:** Number and percentage of various system-specific adverse reactions associated with the administration of taxanes.

Sl. no.	Adverse reactions	Number	Percentage
Dermatologic			
1	Alopecia	40	71.4%
2	Nail discoloration	16	28.5%
3	Mucositis	14	25%
4	Palmar pigmentation	11	19.6%
Hematologic			
1	Anemia	9	19.4%
2	Thrombocytopenia	1	2%
Gastrointestinal			
1	Nausea/vomiting	12	21.4%
2	Diarrhea	5	8.9%
3	Constipation	15	26.7%
Others			
1	Fatigue	30	49%
2	Peripheral neuropathy	31	58.7%
3	Myalgia	25	45.1%

## Discussion

This prospective observational study included 56 patients (mean age: 56 ± 10 years). This study aimed to determine the frequency of adverse reactions to taxanes. This study included 38 male and 18 female participants. Antiemetics (ondansetron), H2 receptor blockers (ranitidine), corticosteroids (dexamethasone), and antihistamines (diphenhydramine) were administered 30 minutes to 1 hour before chemotherapy. In our study, the premedication regimen included ranitidine, an H2 antagonist. However, a study by Huntjens et al. found no significant difference in outcomes between premedication regimens that included ranitidine and those that did not [10]. A study by Villarreal-González et al. found that administering premedications 30 to 80 minutes before the infusion of taxanes in the initial cycles was the optimal timing to reduce the incidence of IRRs [11].

Paclitaxel and docetaxel were the most frequently encountered taxanes in our study for cancers of the breast, ovaries, and endometrium. Our observations regarding the most prescribed taxanes and the types of cancer for which they were prescribed align with those reported in a study by Lai et al. [12].

Our study documented 236 adverse events across all cycles of taxane chemotherapy (206 cycles), with IRRs accounting for only 3.8% (9 out of 236) of all reported adverse events. These results were very similar to those of Roselló et al., who found that IRRs occurred in 2%-4% of premedicated patients with paclitaxel and 2% with docetaxel [13]. However, the percentage of IRRs in patients without premedication was significantly higher (around 30%) in a study by Park et al. [14], thereby underlining the importance of premedication in cancer chemotherapy. Concurrently, it also needs to be noted that certain other studies, incorporating desensitization protocols and Paclitaxel-Hypersensitivity Reaction (Pac-HSR) scoring system for risk factor assessment, have shown a further reduction in the incidence and severity of hypersensitivity reactions and 100% success rate in the completion of taxane therapy [11,15]. Chromophores, which are present in paclitaxel formulations, are a major cause of these reactions. The use of albumin-bound paclitaxel (nab-paclitaxel), which is free of chromophores, can significantly reduce the incidence of these reactions [14]. Consistent with the findings of Tsao et al., the most common IRRs in our study were flushing, dyspnea, and chest tightness [2,5]. However, in a similar study by Trager et al., the most common infusion reactions were maculopapular rash and urticaria [5]. Among the monitored cycles, IRRs were commonly observed during the initial couple of cycles with a severity of grade 2, which was consistent with the findings of Barroso-Sousa et al. [4]. Similarly, our observations were consistent with a study by Xiao et al., which reported that IRRs occurred in 4.5% of patients receiving paclitaxel, with most reactions occurring during the early treatment cycles [16]. These reactions are predominantly IgE mediated, and the development of tolerance may explain their absence in later cycles.

The non-IRRs observed in our study were categorized as dermatologic, abdominal, hematologic, or other adverse events. Dermatologic adverse reactions, such as alopecia, nail discoloration, oral mucositis, and palmar-plantar pigmentation, accounted for a higher proportion of the total adverse reactions recorded. This finding is consistent with the results reported by Sibaud et al. and Trager et al., who also observed a greater number of dermatologic adverse events [5,17]. Cytotoxicity to keratinocytes and disruption of normal cell division may be the underlying mechanisms of these dermatologic adverse reactions.

Abdominal symptoms characterized by nausea, vomiting, diarrhea, and constipation, as well as hematologic events such as anemia and thrombocytopenia, observed in our study, were consistent with the findings of a study by Akbarali et al. [18]. Similar to the study results of Lai et al., our research revealed a lower rate of febrile neutropenia (1% of patients) and thrombocytopenia (2% of patients) as all of our patients on docetaxel received prophylactic granulocyte colony-stimulating factor (G-CSF) [12]. This highlights the importance of regular blood monitoring and prompt administration of hematopoietic growth factors as early indications of bone marrow toxicity. Other adverse events included peripheral neuropathy, fatigue, and myalgia. The findings were in line with the study results of Sibaud et al. [17]. 

Twenty-five participants in our study had comorbid conditions, such as hypertension, dyslipidemia, and diabetes. Although statistically insignificant, neuropathy was more frequently observed in patients with diabetes, and tiredness was reported more frequently in patients on concomitant chemotherapeutic medications. Age and diabetes are independent predictors of peripheral neuropathy, and certain study conclusions are consistent with this fact [19]. 

Similar to IRRs and study results of Sibaud et al., non-IRR adverse events were also observed more frequently during the initial cycles [17]. Non-IRRs that occurred with taxanes also had a common grade 2 severity. All patients were able to complete their treatment cycles without discontinuation, which is consistent with the observations of Villarreal-González et al. [11]. Among the 56 patients across 206 chemotherapy cycles, only one patient with febrile neutropenia required hospitalization, indicating a favorable safety profile of taxanes. 

Using the WHO-UMC scale for causality assessment, we determined that 78% of the ADRs were probable. A higher percentage of probable causality was also found in a similar study on taxanes by Begum et al. [20]. Meanwhile, 22% of ADRs were classified as possible, likely due to the administration of concomitant medications.

Our study has several limitations. First, causality assessment challenges arose because patients received taxanes in combination with other chemotherapy agents, making it difficult to attribute specific adverse reactions solely to taxanes. Second, a limited sample size or short duration of follow-up reduced our study’s ability to detect rare or delayed adverse events, potentially underestimating the true incidence of adverse reactions. Lastly, generalizability was a concern; results obtained from a single center, region, or a specific subset of patients in our study may not have been applicable to broader populations or diverse clinical settings.

## Conclusions

The adverse reactions observed with taxanes in our study indicate that premedication before chemotherapy plays a crucial role in reducing IRRs. The low incidence of IRRs highlights the effectiveness of this preventive strategy. Our findings support the routine use of premedication to enhance patient safety during taxane-based chemotherapy. This approach also helps minimize treatment interruptions and therapy modifications. Overall, maintaining such preventive measures contributes to improved treatment continuity and patient outcomes.
